# Visual Attention to Evolutionarily Relevant Information by Heterosexual Men and Women While Viewing Mock Online Dating Profiles

**DOI:** 10.1007/s10508-024-02950-1

**Published:** 2024-07-15

**Authors:** Madeleine Gale, Rosemary Torbay, Amy D. Lykins

**Affiliations:** 1 School of Psychological Sciences, College of Engineering, Science and Environment, University of Newcastle, Newcastle, NSW Australia; 2 School of Psychology, Faculty of Medicine and Health, University of New England, Psychology Building, Armidale, NSW 2351 Australia

**Keywords:** Visual attention, Eye-tracking, Mate choice, Evolutionary psychology

## Abstract

The way people create social connections and access information has been altered greatly by technology in recent decades. Online browsing of visual profiles has become a common means for seeking potential partners for both short- and long-term relationships. Little is known, however, about how people prioritize mate quality information while viewing online profiles. Using eye-tracking methods and self-report, this study investigated how people evaluated profile-based facial attractiveness and text-based financial resources information, represented by income and occupation. Heterosexual male and female participants, aged between 18 and 27 years, viewed opposite-sex profiles while their eye-movements were recorded using a remote eye-tracking camera. In line with current theory, resources information had little effect on men’s overall attention to women’s faces, whereas women’s overall attention to men’s faces varied depending on the level of income and occupation. Women evaluated men’s faces more when income and occupation were low, regardless of attractiveness. Unexpectedly, however, men marginally increased their attention toward unattractive women who showed a high-level of income and more esteemed occupation. Men self-reported a higher interest in women for a short-term relationship and women self-reported a higher interest in men for a long-term relationship. This work provides a foundation to further examine how people browse profile-based information and to investigate the mate selection process, with real-world implications for online dating app users, profile design, and content.

## Introduction

As technology has become commonplace within society, once traditional in-person activities have gradually transitioned to cyberspace (Stoicescu, 2019). Friends and family once provided a vetting system regarding potential partners; however, online dating profiles now replace this process (Finkel et al., 2012; Rosenfeld et al., 2019). The profile itself communicates the physical characteristics of the potential partner, from which personality attributes and cues regarding potential partner quality are inferred (McGloin & Denes, 2018). Online dating profiles provide immediate and rapid access to a large number of potential mates to pursue or reject, based on the images and personalized text-based information provided in these profiles and their alignment with the individual’s relationship goals (Fiore et al., 2008, 2010; Rosenfeld et al., 2019). It is already well established that individuals preferentially place importance on characteristics that align with their relationship goals (Buss & Schmitt, 2019; Searcy, 1982). However, little is known about how online profile information, including pictures and text, influences impression formation, or how men and women prioritize mate quality information when viewing opposite-sex visual profiles. Furthermore, the relationship between visual attention and self-report, regarding mate choice assessments, using pictures and text, is currently unknown.

### Mate Selection

The manner in which people select and pursue potential mates based on their relationship goals has long been of scientific interest. The characteristics and attributes that an individual seeks can differ based on a multitude of factors, including the individual’s gender and whether they are pursuing a short- or long-term relationship (Buss & Schmitt, 2019). Schmitt (2014) suggests men are more motivated to pursue short-term relationship strategies than women, and that women are more motivated to pursue long-term relationship strategies than men. These findings are in line with both parental investment theory (PIT; Trivers, 1972) and the influential evolution-based sexual strategies theory (SST; Buss & Schmitt, 1993).

Further research supporting PIT and SST suggests that in contrast to men, women are more likely to prioritize cues related to resources (Buss & Schmitt, 1993; Schmitt, 2014; Trivers, 1972) and are more willing to weigh up mate quality, such as ambition and income, with physical attributes (Townsend & Levy, 1990; Townsend & Wasserman, 1998). In determining the suitability of potential male partners for a long-term relationship, women are willing to accept lower levels of attractiveness if the man has greater access to resources. However, women may still prioritize physical attractiveness when seeking short-term relationships. Comparatively, when men select potential mates, physical attractiveness in women far outweighs preferences for high resource status (Townsend & Levy, 1990; Townsend & Wasserman, 1998).

### Online Dating in Heterosexual Men and Women

Deciphering the manner in which individuals select potential mates, especially through the means of online dating profiles, is increasingly relevant given online dating is now commonplace (Stoicescu, 2019). Self-report methodology, in combination with hypothetical dating profiles (using picture and text-based stimuli), generally has been used to examine the evaluation of mate cues in prior research. Studies supporting SST and PIT have found that men are more active on dating sites, less selective regarding potential partners, and more likely to pursue short-term partnerships than women (Abramova et al., 2016). Furthermore, women are more likely to base their potential partner decisions on socio-economic characteristics, in contrast to men, who predominately base decisions on physical appearance (Abramova et al., 2016). Sritharan et al. (2010) reported that women spontaneously evaluate attractiveness, confirming the face as a highly accessible source of information. Furthermore, attractiveness also influences deliberate evaluations when information is consistent (i.e., high attractiveness/high ambition; low attractiveness/low ambition), rather than inconsistent (i.e., low attractiveness/high ambition; high attractiveness/low ambition). That is, spontaneous responses (including evaluation of facial attractiveness) may influence deliberate evaluations (including information relating to ambition) if there is consistency between the attractiveness and ambition.

When viewing dating profiles, participants report that facial images are the strongest predictor of profile attractiveness; however, both the image and the text regions are relevant (Fiore et al., 2008, 2010). After viewing a profile containing a picture of a face, men expressed a strong desire to contact a woman of average attractiveness (Mierke et al., 2011) and 69% of men and 62% of women are willing to become familiar with attractive opposite-sex targets (Bak, 2010). Thus far, self-report methods have been used widely to examine the self-reported preferences of men and women. However, such methods are ill-equipped to measure people’s spontaneous evaluation of information, due to the inherent problems of introspective inability and response bias (Barker et al., 2002). Thus, the decisions that individuals make in selecting partners is well known; however, *how* these decisions are made from a cognitive perspective has received less attention.

### Visual Attention and Dating Profiles

Eye-tracking methodology provides a means with which to assess how decisions regarding potential partners are made, potentially revealing the most influential characteristics in this decision-making process. This methodology allows for a direct, unobtrusive measurement of participants’ spontaneous assessment and prioritization of mate quality cues via the direct recording of eye movements and, thus, visual attention (Holmqvist et al., 2011). Recent eye-tracking studies have found that both pictures and text influence impression formation when assessing romantic attraction.

Dewall and Maner (2008) showed participants photographs of an individual categorized as high attractiveness-high social status, high attractiveness-low social status, average attractiveness-high social status, and average attractiveness-low social status. It was found that high-status men captured 54% of participants’ attention within the four second viewing period, compared to 42% regarding high-status women, and men and women’s attention were similarly directed toward attractive men (59%) and attractive women (59%). Furthermore, when presented with images and text depicting hypothetical dating profiles, pictures attracted initial visual attention, with images depicting highly attractive individuals capturing attention at a higher frequency and for a longer duration compared to those of less attractive individuals (Van der Zanden et al., 2022). While visual attention to photographs and text information in hypothetical online dating profiles is beginning to be investigated, visual attention in the overall assessment of potential mates has received little consideration in literature.

### Current Study

Research into how heterosexual men and women spontaneously allocate their visual attention to mate quality cues (i.e., physical attractiveness and resource potential) is underrepresented in the empirical literature, particularly within the contemporary context of online personal profiles. To date, the predominant methodology used to examine men and women’s stated mate preferences has been heterosexual college-age participants (age range: 18–27 years), self-reporting their mate preferences after viewing experimentally varied visual mate quality cues (Schmitt, 2014). Comparatively, using eye-tracking to record visual attention to dating profiles provides an ecologically valid design and method to study people’s browsing patterns, distinguish what visual information people preference, and quantify and measure mate quality cues.

Thus far, little is known regarding how participants attend to text-based resources *and* physical attractiveness information in profiles under time-limited conditions. This prospect raises new avenues for scientific enquiry into how men and women prioritize two of the most widely studied cues of mate quality, physical attractiveness and resources access, in the context of online dating profiles. Eye-tracking is a methodology capable of testing how people prioritize these cues. Assessing the malleability of men and women’s evolved mate preferences during early mate selection has real-world implications for early impression formation in online dating, profile design, and profile optimization. Together with the wider mate selection research, this study provides a novel line of research that will contribute to a fuller understanding of the earliest stages of information processing involved in the mate selection process.

Thus, the aim of the present study was to measure heterosexual individuals’ initial attention to and self-reported assessment of the different profile regions of a mock online dating profile, while levels of mate quality, such as physical attractiveness and resources access, were manipulated. Based on past literature, the following hypotheses were tested:

1. As the face is a salient visual cue of mate quality with respect to physical attractiveness (Buss, 1989; Buss & Schmitt, 1993; Schmitt, 2014), even when viewed under time limitations (Dewall & Maner, 2008; Sritharan et al., 2010), it was hypothesized that the face region in the profiles would attract the attention of both men and women more so than the resource region.
2. Robust research in support of SST and PIT suggests women are likely to investigate cues to resources more vigorously than men (Buss & Schmitt, 1993; Schmitt, 2014; Trivers, 1972). Women have also expressed their willingness to weigh up mate quality (Townsend & Levy, 1990; Townsend & Wasserman, 1998), for example, by compensating men’s high resources for lower-level attractiveness and high attractiveness for lower-level resources. On this basis, the second hypothesis predicted that women’s attention to the face region in men’s profiles—and thus men’s physical attractiveness—would vary depending on the target’s level of income and occupation.
3. Consistent with SST (Buss & Schmitt, 1993), physical attractiveness is found to far outweigh men’s preference for women’s resource potential (Dunn & Hill, 2014; Dunn & Searle, 2010; Townsend & Levy, 1990; Townsend & Wasserman, 1998). Therefore, the third hypothesis predicted that men’s attention would be directed toward women’s faces, particularly attractive faces, irrespective of income and occupational status.
4. Lastly, as self-reported assessments of mate quality have been shown to be consistent with SST and PIT (Abramova et al., 2016), it is predicted that men would report highly attractive women as more attractive for a short-term relationship compared to a long-term relationship, irrespective of resources, and women would report high resource men as more attractive for a long-term relationship compared to a short-term relationship, irrespective of physical attractiveness.

## Method

### Participants

G*Power analysis (Faul et al., 2009) was undertaken to determine the sample size required to conduct an analysis of variance (ANOVA), with testing of interactions between and within groups. Assuming 80% power and a medium effect size (η^2^ = 0.06; Cohen, 1988), the analysis recommended 24 participants. Previous eye-tracking studies investigating attention to sexually attractive adult men and women have found large effects of η^2^ = 0.41 (Lykins et al., 2006) and η^2^ = 0.73 (Lykins et al., 2008) for ANOVA interactions in samples of 40 participants. To secure acceptable power in line with previous studies, we aimed to recruit 20 men and 20 women who provided high-quality data.

Fifty-five participants were recruited via poster, flyer and relevant online forums within the university. Inclusion criteria for participation were as follows: (1) 18 years of age or older; (2) heterosexual-identified; (3) identity of male or female; and (4) normal or corrected-to-normal vision. Fifteen of the 55 participants were excluded from the study for the following reasons: (1) four presented with ocular and/or eye-movement deficits; (2) six had incomplete eye-movement data; and (3) five participants were not included in the age-matched sample.

The final sample consisted of 20 men (*M*_age_ = 20.85, *SD* = 2.35; age range: 18–27 years) and 20 women (*M*_age_ = 20.10, *SD* = 2.29; age range: 18–27 years). All participants were university students, about half of whom received research course credit for their participation; no other incentives for participation were offered. Seventy-five percent of participants reported a relationship status of single, and 25% reported being in a relationship. All participants reported an annual income of $0–$30,000, placing them in the lowest income band as per the Australian Bureau of Statistics (2015). Participants provided written informed consent prior to commencing the study.

### Apparatus

An Applied Sciences Laboratories Eye-Trac D6 desk mounted optics system measured participants’ right eye movements at a sampling rate of 120 Hz with 0.5-degree visual angle accuracy. The Eye-Trac D6 uses the reflection measurement principle to capture eye-movements by correlating the *x*- and *y*-coordinates of the pupil and cornea to where the eye looks on the stimulus display. Gaze Tracker (Version 9; Lankford, 2000) was used to create and present the stimuli and to record eye data. The stimuli were viewed on a Dell UltraSharp Monitor (1920 × 1200 pixels; 61 cm screen; 16:10 aspect ratio).

### Stimuli Selection

The visual stimuli comprised facial images representing different levels of physical attractiveness (high, low) and different levels of text-based demographics indicating resource potential (high, low). The facial images were sourced from existing databases and collections (Centre of Vital Longevity, 2008; Psychological Image Collection at Stirling, 2003; Rupp et al., 2009). The black and white images were adjusted for luminance levels and size (800 × 600 pixels) to achieve uniform values.

A total of 72 facial images of men and women were pre-rated for attractiveness via an online pilot questionnaire distributed to heterosexual off-campus university students. Twenty men (*M*_age_ = 32.95, *SD* = 9.24) and 20 women (*M*_age_ = 34.10, *SD* = 9.74), who did not participate in the main study, rated opposite sex (with respect to the participant’s identified gender) faces from 0 (*very unattractive*) to 7 (*very attractive*). For both men and women, the 12 top-rated faces were selected to represent high attractiveness and the 12 lowest rated faces were selected to represent low attractiveness. This resulted in 24 facial images of men (lowest rating: *M* = 2.62, *SD* = 0.32; highest rating: *M* = 4.54, *SD* = 0.30) and 24 of women (lowest rating: *M* = 2.65, *SD* = 0.38; highest rating: *M* = 5.27, *SD* = 0.31). The high and low levels of resource potential and occupation were represented by the highest average and the minimum Australian wages and associated occupations (Australian Bureau of Statistics, 2015; Australian Fair Work Commission, 2015; PayScale Australia, 2015).

### Profile Stimulus Design

**Fig. 1:**
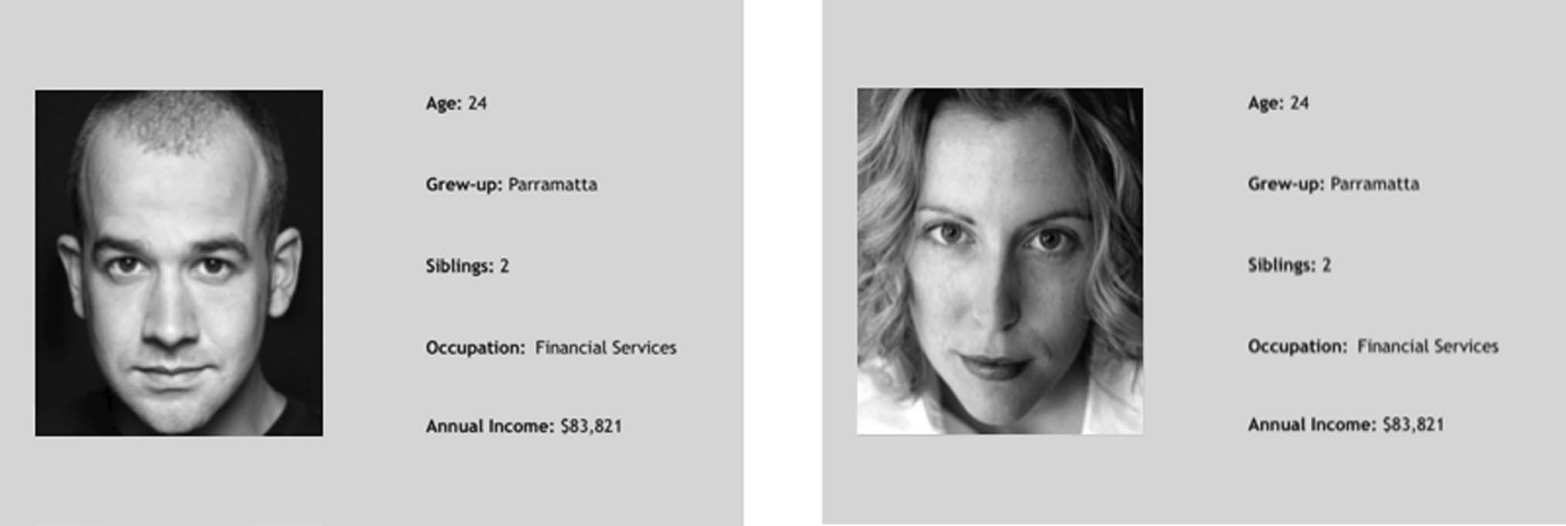
Example of a male and female profile template. Note that these examples were not among the final experimental stimuli. Photos from Rupp et al. (2009)

The stimuli were standardized using a profile-like template measuring 40 cm × 28 cm. One facial image and one text-based demographic component, both measuring 15 cm × 8 cm, were positioned side-by-side within the template (see example in Fig. 1). Twenty-four text-based components were constructed to include neutral demographic information (i.e., “Age” [ranging: 18–33 years to match approximate age span of participants], “Grew up” [regional and metropolitan towns/cities] and “Siblings” [numbered: 0–4] as filler information). Either high or low income and occupational information was then added. To control for variation between men and women’s text-based information, the final 24 demographic profile components were duplicated so that men and women participants would view the same compilation of demographic information. Attractive or unattractive facial photographs of men and women were added to the profiles to determine the target’s sex and level of physical attractiveness.

**Fig. 2:**
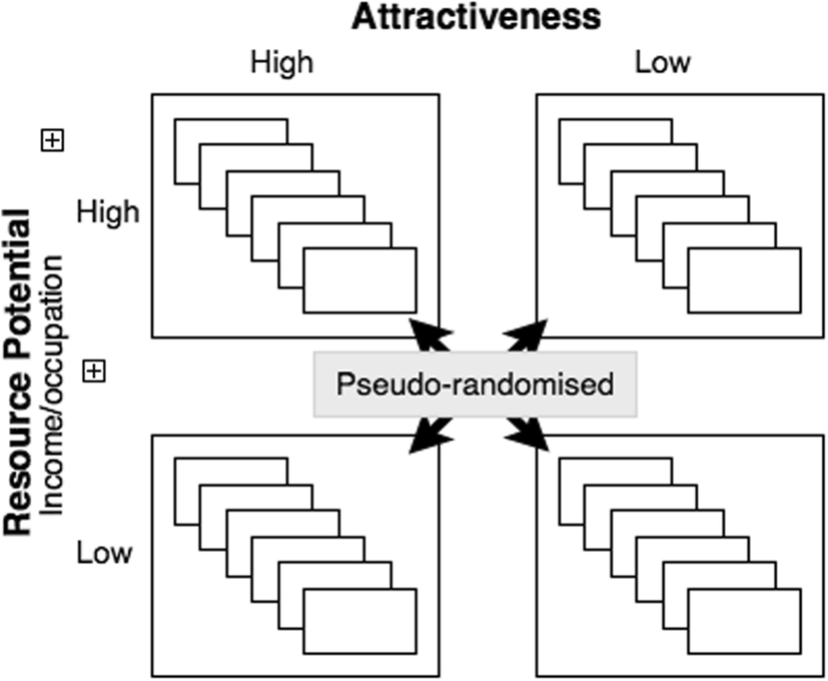
Illustration showing how profile conditions were created. Each stimuli block contained 24 profiles representing all possible combinations of attractiveness (high, low) and resource potential (high, low). The profiles within the block were pseudo-randomised

In the final set of stimuli, each stimulus image included one photograph of a face (high or low attractiveness) combined with a text-based box including the filler demographic information as described above, along with information about the individual’s income and occupation (high or low income/occupation). This resulted in four stimulus conditions: high attractiveness, high resources; high attractiveness, low resources; low attractiveness, high resources; and low attractiveness, low resources. These four profile conditions were presented in 6 different unique profiles for both men and women, resulting in a total of 24 unique profiles of men and 24 unique profiles of women. The profiles were pseudo-randomized to minimize successive presentation of same conditions (see Fig. 2).

A gray slide featuring a central black dot was inserted before each profile. This provided participants with a neutral fixation point prior to viewing each profile, thus reducing unintentional visual bias toward the face or the resource region. In addition, the fixation point provided researchers with a calibration checkpoint between slides.

#### Research Design

A quasi-experimental mixed design was employed to analyze the data. Sex of participant (male vs. female) was the between-subjects factor and scene-region (face vs. resources), attractiveness (high vs. low), and resource potential (high vs. low) were the within-subjects factors. Using Gaze Tracker, each profile was separated into two underlying regions of interest (ROIs): the face and resource regions. Eye movements to these regions were recorded.

The eye-movement measure of total gaze time used in this study has been previously linked to attention across a variety of tasks (for reviews see, Liversedge & Findlay, 2000; Rayner, 1998). Total gaze time is a recognized measure of overall attention to ROIs within the visual stimuli (Holmqvist et al., 2011), with longer gaze time indicating greater interest (Henderson & Hollingworth, 1999).

### Procedure

Participants individually attended the eye-tracking laboratory, a quiet room with controlled lighting. After reading the information sheet and providing informed consent, each participant received a briefing on the eye-tracking procedure. Once participants were seated at a viewing distance of 60 cm from the computer monitor, they undertook a Snellen eye test to confirm satisfactory visual acuity. Participants then underwent a standard 9-point calibration procedure to confirm that their eye movements were being recorded accurately.

**Fig. 3:**
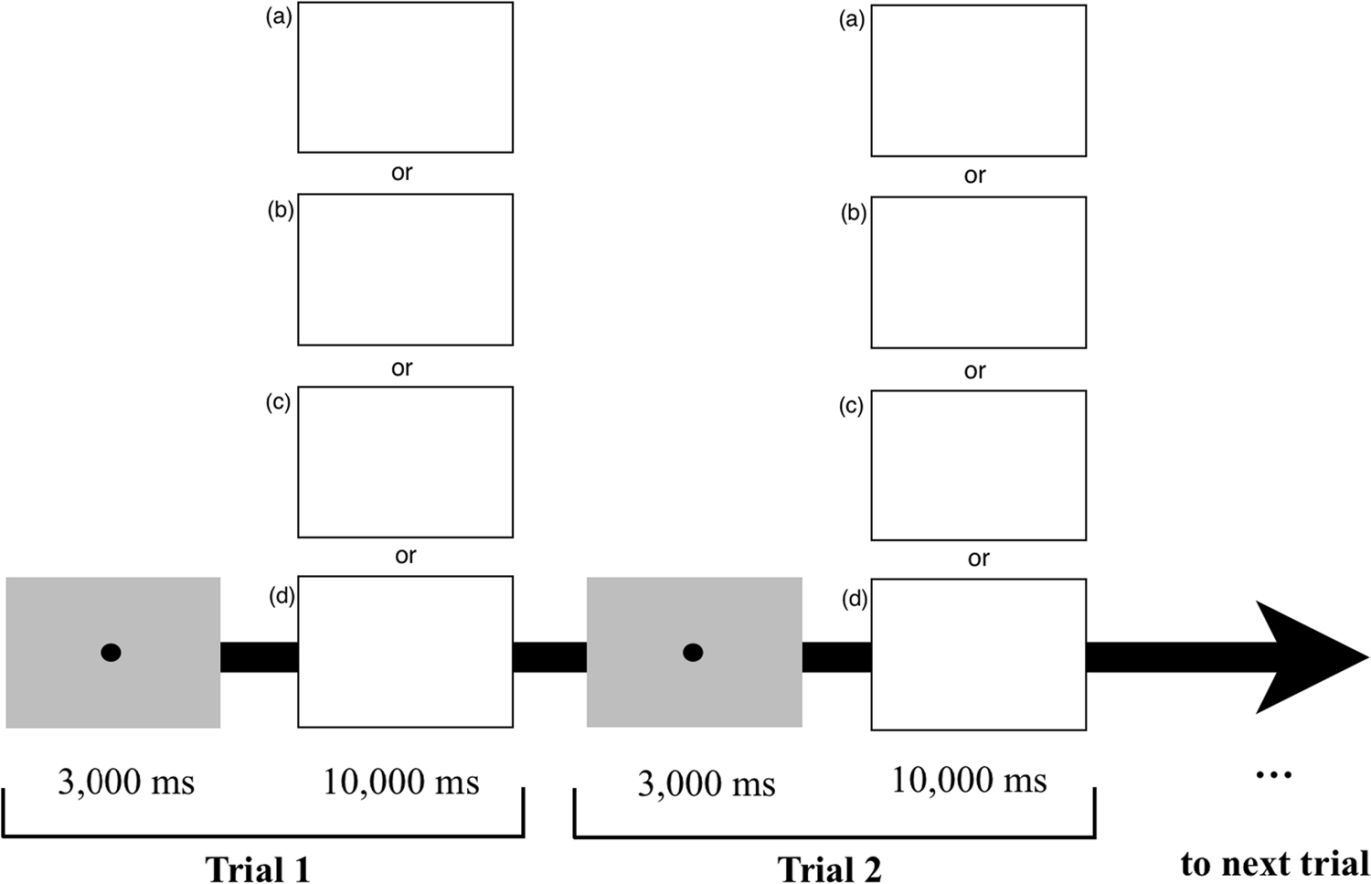
Illustration of two experimental trial showing the time sequence. One of four possible opposite-sex profiles was presented at each trials: **a** attractiveness (high) and income/occupation (high), **b** attractiveness (high) and income/occupation (low), **c** attractiveness (low) and income/occupation (high), or **d** attractiveness (low) and income/occupation (low)

Participants were told to “observe the profiles as you would normally.” Once the experimental session commenced, participants viewed the 24 opposite-sex profiles consecutively. To control for stimulus presentation time across participants, each profile remained on the screen for 10,000 ms with the calibration dot slide displayed for 3,000 ms prior to each profile (see Fig. 3). After the presentation of each slide, participants were asked to rate the person in the image on their overall level of attractiveness, and their levels of attractiveness for short- and long-term relationships, respectively, on a 7-point Likert scale from 1 (very unattractive) to 7 (very attractive). Participants simply clicked a number on the screen using the mouse to provide this information so that the eye-tracking calibration was not lost in between stimuli.

Female participants viewed the same block of profiles featuring men’s faces, and men, the same block of profiles featuring women’s faces. The duration of the eye-tracking session was approximately 5 min. Participants then completed a basic demographic questionnaire to collect data on age, gender, relationship status, income, and education. Course credit paperwork was also completed if applicable to the participant.

## Results

### Statistical Analyses

The aim of the current study was to assess how men and women visually prioritize cues of mate quality, in particular physical attractiveness and resource potential. Prioritization of information was measured by total gaze time: the amount of time spent viewing the different mate quality cue ROIs. Eye movements were recorded and aggregated by Gaze Tracker, with over 240,000 data points then exported into an Excel spreadsheet using custom-built macro programs. Finally, for each participant, the mean total gaze times within two scene regions (face vs. resources) were calculated across profile conditions.

To test men’s and women’s visual attention to the different profile regions and toward varying levels of physical attractiveness and income/occupation, total gaze time data were analyzed in a 2 (sex of participant: male vs. female) × 2 (attractiveness: high vs. low) × 2 (resource potential: high income and occupation vs. low income and occupation) × 2 (scene region; face vs. resources) mixed-design ANOVA using SPSS (v. 29). In order to analyze self-reported attractiveness and attractiveness for different types of relationships, a 2 (sex of participant: male vs. female) × 2 (attractiveness: high vs. low) × 2 (resource potential: high income and occupation vs. low income and occupation) × 3 (self-reported: attractiveness vs. short-term relationship vs. long-term relationship). Interactions reaching significance were further evaluated with simple effects analysis using the COMPARE method on the EMMEANS subcommand in SPSS. Each pairwise comparison was treated as its own family for the purposes of Type 1 error correction (IBM, 2010).

### Overview of Main Analyses

Means and standard deviations for men’s and women’s total gaze time to each of the profile ROIs are shown in Table 1. Results for a four-way mixed ANOVA testing total gaze time to profile-based mate quality cues are presented in Table 2. Two significant main effects were found (Scene Region, Resource Potential), as were two significant two-way interactions (Scene Region × Resource Potential, Sex × Resource Potential) and two three-way interactions (Sex × Scene Region × Resource Potential, Sex × Attractiveness × Resource Potential). The four-way Sex × Scene Region × Attractiveness × Resource Potential did not reach significance. Table 3 shows men’s and women’s means and standard deviations for self-report data.

**Table 1:** Mean scores and standard deviations for total gaze times to profile conditions

	High Attractiveness + High Resources		High Attractiveness + Low Resources		Low Attractiveness + High Resources		Low Attractiveness + Low Resources	
Participants	*M*	*SD*	*M*	*SD*	*M*	*SD*	*M*	*SD*
*Female*								
Face	5.29	0.98	5.51	1.03	5.04	0.91	5.55	0.97
Resources	1.10	0.54	1.08	0.48	1.28	0.35	1.12	0.42
*Male*								
Face	5.34	1.27	5.43	1.30	5.43	1.46	5.23	1.35
Resources	0.95	0.77	1.05	0.82	1.05	0.83	0.93	0.77
*Both groups*								
Face	5.32	1.12	5.47	1.16	5.24	1.21	5.39	1.17
Resources	1.02	0.66	1.06	0.66	1.16	0.71	1.02	0.62

**Table 2:** Analysis of variance on total gaze time for 4-way interaction (Sex of Participant × Attractiveness × Resource Potential × Scene Region)

Source	*df*	*SS*	*MS*	*F*	*η*_*p*_^*2*^	*p*
		Between subjects				
Sex of participant (S)	1	0.39	0.39	0.18	0.01	0.67
G within-group error	38	82.74				
		Within subjects				
*Main effects*						
Attractiveness	1	0.02	0.02	0.19	0.01	0.66
Resource Potential	1	0.21	0.21	4.09	0.10	0.05
Scene Region	1	1469.76	1469.76	341.03	0.90	< 0.001
*Two-way interactions*						
A × RP	1	0.16	0.16	2.12	0.05	0.15
SR × A	1	0.31	0.31	1.32	0.03	0.26
S × A	1	0.02	0.02	0.25	0.01	0.62
SR × RP	1	0.83	0.83	6.22	0.14	0.02
S × RP	1	0.59	0.59	11.74	0.24	< 0.001
S × SR	1	0.49	0.49	0.11	0.003	0.74
*Three-way interactions*						
S × SR × A	1	0.15	0.15	0.62	0.02	0.44
S × SR × RP	1	1.24	1.24	9.31	0.20	0.004
S × A × RP	1	0.54	0.54	7.16	0.16	0.01
SR × A × RP	1	0.16	0.16	0.77	0.02	0.39
*Four-way interactions*	1					
S × SR × A × RP	1	0.30	0.30	1.42	0.04	0.24

S = Sex of participant, A = Attractiveness of profile image, RP = Resource potential, SR = scene region

**Table 3:** Mean scores and standard deviations for total gaze time to profile conditions

	High Attractiveness + High Resources		High Attractiveness + Low Resources		Low Attractiveness + High Resources		Low Attractiveness + Low Resources	
Participants	*M*	*SD*	*M*	*SD*	*M*	*SD*	*M*	*SD*
*Female*								
Short-term	4.80	0.73	4.23	0.90	2.13	0.59	2.50	0.61
Long-term	4.83	0.77	4.18	0.88	2.61	0.66	2.77	0.62
Attractiveness	4.80	0.61	4.52	0.78	2.60	0.50	2.93	0.50
*Male*								
Short-term	4.78	0.87	5.02	0.89	3.12	1.13	2.28	0.86
Long-term	4.68	1.02	4.53	0.97	3.18	1.18	2.03	0.78
Attractiveness	5.03	0.73	5.09	0.69	3.33	1.06	2.38	0.79
*Both groups*								
Short-term	4.68	0.80	4.63	0.97	2.62	1.02	2.39	0.74
Long-term	4.75	0.89	4.36	0.93	2.90	0.99	2.40	0.79
Attractiveness	4.91	0.68	4.80	0.78	2.97	0.90	2.65	0.71

### Eye-Tracking Data Analyses

For total gaze time, the results indicated significant main effects for Scene Region, *F*(1, 38) = 341.03, *p* < 0.001, η_p_^2^ = 0.90, and Resource Potential, *F*(1, 38) = 4.09, *p* = 0.050, η_p_^2^ = 0.10. These main effects were qualified by a significant interaction of Scene Region × Resource Potential, *F*(1, 38) = 6.22, *p* = 0.017, η_p_^2^ = 0.14. This effect was due to participants looking longer at the face region when income and occupation were low (vs. high) irrespective of participant sex, *F*(1, 38) = 6.76, *p* = 0.013, η_p_^2^ = 0.15, 95% CI [0.03, 2.72].

**Fig. 4:**
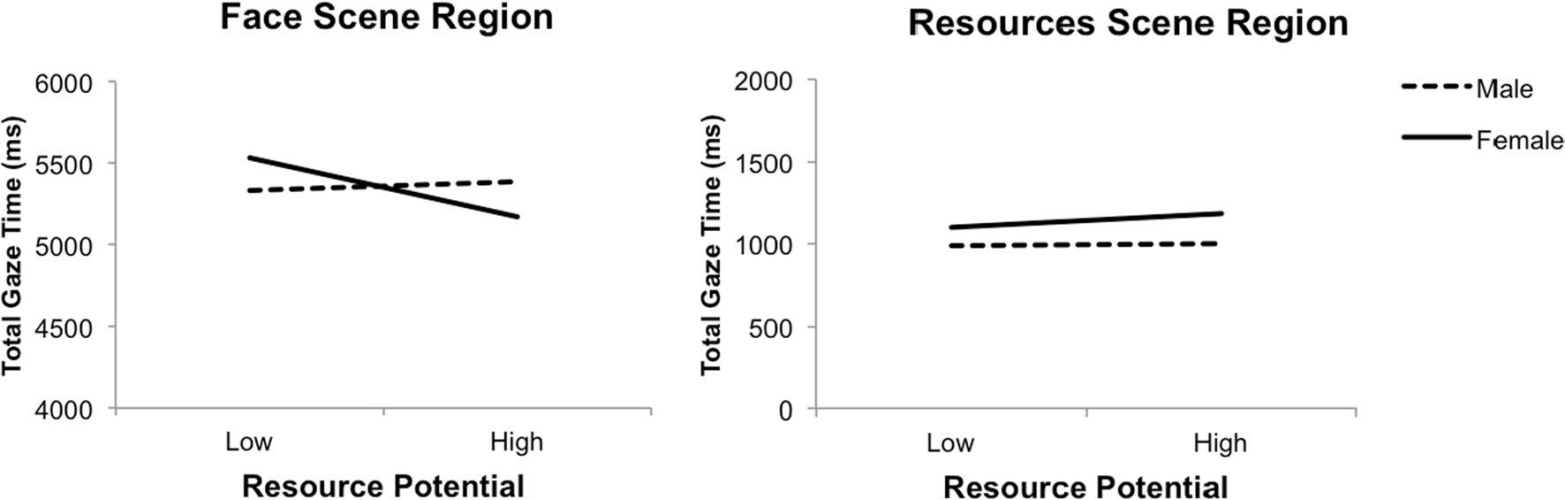
Three way interaction of Sex × Scene Region × Resource Potential for total gaze time

A significant interaction of Sex × Resource Potential, *F*(1, 38) = 11.74, *p* = 0.001, η_p_^2^ = 0.24, clarified the effect of low income and occupation. Women were found to look at low (vs. high) income and occupation for longer periods, *F*(1, 38) = 14.84, *p* < 0.001, η_p_^2^ = 0.28, 95% CI [0.07, 0.21]. A significant interaction of Sex × Resource Potential × Scene Region, *F*(1, 38) = 9.31, *p* = 0.004, η_p_^2^ = 0.20, captured the abovementioned main effects and interactions (see Fig. 4). Taken together, the results indicated that: (1) men and women spent more time overall looking at the face (vs. resources) region; (2) men and women looked similarly at the resource region, regardless of the level of income and occupation; (3) on average, men and women spent similar time looking at faces; (4) when income and occupation were low (vs. high), women looked significantly longer at men’s faces, *F*(1, 38) = 19.13, *p* < 0.001, η_p_^2^ = 0.34, 95% CI [0.20, 0.53]; and (e) the levels of income and occupation did not significantly affect how long men looked at women’s faces, *F*(1, 38) = 0.99, *p* = 0.327, η_p_^2^ = 0.03.

**Fig. 5:**
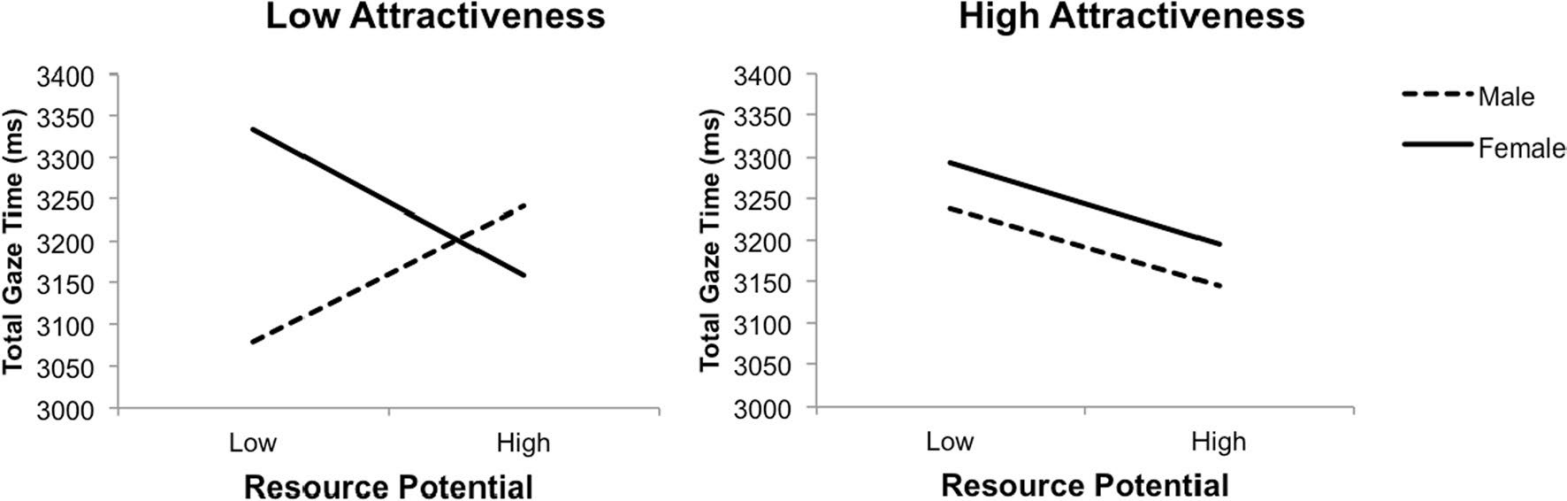
Three-way interaction of Sex × Attractiveness × Resource Potential for total gaze time

A significant interaction of Sex × Attractiveness × Resource Potential, *F*(1, 38) = 7.16, *p* = 0.011, η_p_^2^ = 0.16, is illustrated in Fig. 5. The effects indicated: (1) women’s time looking at unattractive men significantly increased when income and occupation were low (vs. high), *F*(1, 38) = 7.80, *p* = 0.008, η_p_^2^ = 0.17, 95% CI [0.05, 0.30]; (2) women’s time looking at attractive men marginally increased when income and occupation were low (vs. high), *F*(1, 38) = 4.01, *p* = 0.052, η_p_^2^ = 0.10, 95% CI [− 0.20, 0.001]; (3) men’s time looking at unattractive women significantly increased when income and occupation were high (vs. low), *F*(1, 38) = 6.81, *p* = 0.013, η_p_^2^ = 0.15, 95% CI [0.04, 0.29]; (4) men’s time looking at attractive women marginally increased when income and occupation were low (vs. high), *F*(1, 38) = 3.50, *p* = 0.069, η_p_^2^ = 0.08, 95% CI [− 0.19, 0.008]; and (5) low (vs. high) income and occupation similarly influenced men and women’s time looking at attractive opposite-sex faces, *F*(1, 38) = 0.12, *p* = 0.731, η_p_^2^ = 0.003.

### Self-Reported Attractiveness Ratings Analyses

For self-report data on ratings of attractiveness, significant main effects were found for Attractiveness, *F*(1, 38) = 413.74, *p* < 0.001, η_p_^2^ = 0.92, Resource Potential, *F*(1, 38) = 15.76, *p* < 0.001, η_p_^2^ = 0.29, and Self-Reported Attractiveness and Attractiveness for a Relationship, *F*(2, 76) = 9.69, *p* < 0.001, η_p_^2^ = 0.20. These main effects were clarified by the significant interactions of Resource Potential × Self-Reported Attractiveness and Attractiveness for a Relationship, *F*(2, 76) = 11.88, *p* < 0.001, η_p_^2^ = 0.24, and Attractiveness × Self-Reported Attractiveness and Attractiveness for a Relationship, *F*(2, 76) = 5.83, *p* = 0.004, η_p_^2^ = 0.13. Regardless of sex, when presented with low resources and low attractiveness stimuli, participants rated opposite sex individuals as higher in attractiveness and higher for a short- or long-term relationship, *F*(1, 38) = 1.40, *p* = 0.244, η_p_^2^ = 0.04.

Significant interactions between Sex × Resource Potential, *F*(1, 38) = 8.70, *p* = 0.005, η_p_^2^ = 0.19, Sex × Self-Reported Attractiveness and Attractiveness for a relationship, *F*(2, 76) = 5.53, *p* = 0.006, η_p_^2^ = 0.13 and Sex × Attractiveness × Resource Potential, F(1, 38) = 44.62, *p* < 0.001, η_p_^2^ = 0.54, were found. Taken together, these results show: (1) men rated women higher for a short-term relationship (*M* = 5.02; *SD* = 0.89) than for a long-term relationship, (*M* = 4.53; *SD* = 0.97) when presented with high attractiveness and low resources stimuli, *F*(2, 37) = 7.32, *p* = 0.002, η_p_^2^ = 0.28; (2) similarly, men rated women higher for a short-term relationship (*M* = 2.28; *SD* = 0.86) than for a long-term relationship, (*M* = 2.03; *SD* = 0.78) when presented with low attractiveness and low resources stimuli, *F*(2, 37) = 7.84, *p* < 0.001, η_p_^2^ = 0.30; (3) comparatively, women rated men higher for a long-term relationship (*M* = 2.61; *SD* = 0.66) than for a short-term relationship, (*M* = 2.13; *SD* = 0.59) when presented with low attractiveness and high resources stimuli, *F*(2, 37) = 14.04, *p* < 0.001, η_p_^2^ = 0.43; and (4) women also rated men higher for a long-term relationship (*M* = 2.77; *SD* = 0.62) than for a short-term relationship, (*M* = 2.50; *SD* = 0.61) when presented with low attractiveness and low resources stimuli, *F*(2, 37) = 12.82, *p* < 0.001, η_p_^2^ = 0.41.

## Discussion

This novel study examined how men and women visually prioritize mate selection information in a contemporary context, as well as how varying attractiveness and resource potential affected self-reported ratings of overall attractiveness, and potential for a short- or long-term relationship. Overall, participants directed more visual attention to the face region as compared to the resource region and spent longer looking at the face region when income and occupation were low. This result supports the first hypothesis. A significant effect of income and occupation on women’s attention to men’s faces provided support for the second hypothesis. Analyses indicated that regardless of facial attractiveness, women spent significantly more time looking at men’s faces when income and occupation were low. The third hypothesis was partially supported, as resources had little effect on men’s overall average attention to women’s faces. However, men’s attention to unattractive women was significantly affected by high income and occupation, and a marginal effect was found where men attended more to attractive women when income and occupation was low. Similarly, the fourth hypothesis was partially supported, as overall, men reported greater interest in women for a short-term relationship compared to a long-term relationship and women overall reported men higher for a long-term relationship compared to a short-term relationship. However, unexpectedly, men rated women with low attractiveness and low resources higher for a short-term relationship compared to a long-term relationship, and women rated men with low resources and low attractiveness higher for a long-term relationship as compared to a short-term relationship.

### Prioritizing Profile Mate Quality: The Face

Men and women exhibited very similar patterns of overall attention toward the face and resource regions. Of the total time spent looking in both regions, 83% was directed to the face region. These results are in line with previous evidence, confirming that the face provides crucial information when viewed in profile-based stimuli (Bak, 2010; Fiore et al., 2008; Hitsch et al., 2010; Mierke et al., 2011; Sritharan, 2010). It is possible that the predictable placement of information in profiles could induce visual bias. However, to mitigate this, the face and text regions were similarly sized, participants were asked to focus on a central fixation point between profiles, and presentation time was controlled across participants. Consistent with previous research, faces draw an inordinate amount of attention when presented visually.

### Prioritizing Profile Mate Quality: Facial Attractiveness, Income, and Occupation

The results indicated that women varied their overall attention toward men’s faces depending on his reported resource potential. In contrast, men’s overall average time looking at women’s faces was only marginally affected by resource information. This finding confirms predictions posed by SST (Buss & Schmitt, 1993) and prior findings based on visual stimuli and self-report methods (Dunn & Hill, 2014; Townsend & Levy, 1990; Townsend & Wasserman, 1998). However, when eye movements to the different levels of income, occupation, and attractiveness were analyzed, unique patterns of visual prioritization emerged.

### How Women Prioritize Physical Attractiveness, Income, and Occupation

In the current study, low income and occupation information had a strong effect on women’s increased attention to both attractive and unattractive men. To determine men’s overall mate quality, eye-movement data suggest that women could be integrating income, occupation, and attractiveness information during the first 10,000 ms. Prior findings suggest that women, more than men, adopt an evaluative, compensatory approach when they preference opposite-sex attractiveness and resources (Townsend & Levy, 1990; Townsend & Wasserman, 1998). In the current study, women compensating for low resources potential with high physical attractiveness in men might partially explain their visual patterns. However, women’s increased attention to unattractive men, who were resource poor, would not be considered a compensatory strategy. If women were expected to weigh up the costs and benefits of a potential mate (Buss, 1989; Buss & Schmitt, 1993; Trivers, 1972), then a compensatory pattern of high/low or low/high for attractiveness and resources would be more likely than a low/low pattern. Women’s reduced attention toward men’s faces when resources were high (vs. low) suggests they could be spontaneously evaluating information when it is consistent with their preferences. However, when the level of resources was found to be inconsistent with women’s preferences (i.e., low), they may have evaluated information more deliberately to weigh-up men’s overall mate quality. With the expectation that women will be more stringent than men in their deliberations of mate quality (Buss, 1989; Buss & Schmitt, 1993; Trivers, 1972), the current findings suggest a process of evaluating the best of what’s available from a pool of available mates.

### How Men Prioritize Attractiveness, Income, and Occupation

Collectively, the current findings suggest that men’s patterns of early visual contemplation toward attractive and unattractive women were motivated by income and occupation information. This is an unexpected finding, due to the well-documented self-reported preference for attractive women and men’s typical lower concern regarding the resource access of potential partners. However, self-reporting of one’s ideal mate preferences is a more deliberate, goal-specific evaluative process, whereas eye-tracking provides a second-by-second account of how mate quality information is discriminated during the initial moments of the first encounter of a potential mate (in this case, her online dating profile).

The current study unveiled a unique pattern, whereby men looked at unattractive women significantly more when income and occupation were high and attended marginally more to attractive women when resources were low. Even though prior research has predominantly found men’s evolved preference is for attractiveness (Schmitt, 2014), women of average attractiveness have also been found to attract men’s attention (Dewall & Maner, 2008; Mierke et al., 2011). Men’s contemplation over unattractive women with resources, however, has not been reported in the mate selection literature to date.

A number of reasons could explain why men differentiated their attention to attractive and unattractive women depending on the level of resources. Men’s attention to unattractive women may have been prompted by the caliber of occupations presented in the high condition, for example, health and safety (mining), medical science liaison, and media services. Unattractive women with higher-level income and occupations may have evoked increased attention from the college-aged participants in the current study who reported earnings less than $30,000 per year. However, high resources had little effect on men’s attention to attractive women. Men’s attention only increased toward attractive women when resources were low. This result could be interpreted as reflecting men’s evolved preferences for attractiveness over resources.

### Self-Reported Assessments of Mate Quality

Participants’ level of interest or difficulty in integrating information cannot be determined by eye-tracking alone. By incorporating self-reporting of participants’ romantic interests alongside eye-tracking, this attention-interest gap was examined further.

Overall, participants reported opposite sex individuals as highly attractive for the presumed relationship length. Supporting prior research, men rated high attractive and low resources profile higher for short-term relationships, and women rated low attractiveness and high resource profiles as higher for long-term relationships. This finding supports stated evolutionary preferences depicted by SST and PIT as well as the study Abramova et al. (2016) conducted, which found that men prioritize attractiveness and women prioritize resources. Men rating women with low attractiveness and low resources higher for a short-term relationship was an unexpected finding. Due to the consistent information (i.e., low attractiveness and low resources), explicit decision-making may have been altered, an interpretation supported by the results of Sritharan et al. (2010). However, women rated men with low resources higher for a long-term relationship, which was also unexpected. For both men and women, decisions may have been made based on the available pool of potential mates, which was inherently more limited than one would find via an actual dating app.

### Limitations and Future Directions

The possibility of gender stereotyping or intellectual inferences due to occupation types may warrant further investigation. Attempts were made to minimize extraneous occupation type effects by selecting gender-neutral occupations for men and women’s profiles. For example, the low resource condition profiles included hospitality, retail services, and telemarketing. More balance between experimental control and ecological validity could be achieved by investigating richer real-world profile design and content. This aim could be achieved through breaking the profiles down into smaller regions, including additional conditions, and manipulating different elements.

Analyzing a wider range of demographic information would account for people’s visual attention to other mate quality information available in profiles. Also, separating income from occupation may help to differentiate how each element affects participants’ attention. Furthermore, a pilot study of income and occupation information among a similar population would confirm its relevance and strength as a representation of resources, particularly among young, educated college-aged students.

In future studies, assessing attention shift between face and text information may be beneficial in order to investigate the manner in which surprising or inconsistent information is dealt with cognitively. It may also be worth investigating other dependent variables of interest, such as initial fixation, to assess early attentional capture. Lastly, a larger sample size may be beneficial, although we note that this study incorporated 240,000 points of data per participant and the methods between this study and studies justifying the sample size were comparable (i.e., free viewing of attractive adult men and women).

### Conclusion

This study provides evidence that eye-tracking can detect differences in visual attention toward the profile-based mate quality cues of physical attractiveness and resource potential (as indicated by income and occupation). During early browsing of profiles, men and women have used novel patterns to prioritize different information presented. Broader-level findings indicated that sex-differentiated mate preferences were consistent with evolution-based theory and prior research. Unexpectedly, however, unattractive women with high resources captured men’s attention, whereas women were found to deliberate differently on men’s faces depending on the level of resources. What men and women say they want in a mate is well established in the literature. This study is the first to narrow the research focus down to the first 10 s of profile browsing to assess what information is visually prioritized. This work has real-world implications for profile users, design, and content, and confirms that implicit and explicit decision-making in an online environment is consistent with the evolutionary theories of SST and PIT. Our results provide a foundation from which to further examine how people browse profile-based information and to assess how people seek, pursue, or reject potential mates.

## Data Availability

Data can be made available by request to the corresponding author.
